# 

**Published:** 1892-12

**Authors:** 


### The Library of the Bristol Medico-Chirurgical Society.

At the Annual Meeting of the Society, held on October 12th, Mr. L. M. Griffiths, the Honorary Librarian, read the following Report:—

Since the Annual Meeting of the Society last year 679 volumes have been added to the Library, which now, including 264 duplicates, contains 2,564 volumes.

The donors of money during that period have been Mr. E. C. Board and Mr. S. H. Swayne. Those who have given books are Dr. Barclay Baron, British Medical Association, Dr. Michell Clarke, Mr. G. G. Corbould, Dr. D. S. Davies, Mr. W. Duncan, Dr. C. Elliott, Mr. H. W. Freeman, Mr. L. M. Griffiths, Dr. Clements Hailes, Mr. W. H. Harsant, Dr. Percy Mathews, Mr. J. H. Norman, Mr. C. F. Pickering, Dr. Arthur B. Prowse, Dr. J. E. Shaw, Mr. Greig Smith,

Dr. Shingleton Smith, Mr. Simeon Snell, The Staff of St. George's Hospital, Dr. Charles Steele, The Surgeon-General United States Army, Dr. J. G. Swayne, Dr. F. J. Wethered and Dr. Watson Williams.

Many additions are required to complete sets of books, more especially of periodicals. Towards this every member of the Society might do something. An odd volume, or even an odd number, would often be acceptable.

It has been suggested that our Library should be amalgamated with that of the Medical School, and that our books should be housed in the new School Buildings. For the consideration of this suggestion your Committee at a recent meeting appointed three of its members to talk over the matter with three members of the Faculty of the Medical School and report the result. The conference has not yet taken place, and now would be a convenient time for members of the Society generally to express their views of the proposal.

If the amalgamation should take place, doubtless those members of the Society, who have been withholding their contributions till a larger scheme should be developed, will then feel themselves unfettered in the matter of adding to the Society's store of books or of enriching our own Library Fund.

---

*The following donations have been received since the publication of the List in September:*

*November* 30*th*, 1892.

| | £ | *s.* | *d.* | |
|---|---|---|---|---|
| British Medical Association (1) ... | | | | ... 221 volumes. |
| Alexander Carr (2) ... ... ... | | | | ... 1 volume. |
| Percy Mathews, M.D. (3) ... | | | | ... 1 ,, |
| Arthur B. Prowse, M.D. (4) ... | | | | ... 1 ,, |
| The Staff of St. Thomas's Hospital (5) ... ... ... | | | | ... 18 volumes. |
| J. Greig Smith (6) ... ... ... | | | | ... 2 ,, |
| R. Shingleton Smith, M.D. (7)... | | | | ... 1 volume. |
| Charles Steele, M.D. (8)... ... | | | | ... 12 volumes. |
| Henry Waldo, M.D. ... ... ... | 5 | 5 | 0 | |
| F. J. Wethered, M.D. (9) ... | | | | ... 1 volume. |

Unbound periodicals have been received from the British Medical Association, Dr. Prowse, and Dr. Steele.

Framed pictures have been presented by Dr. Michell Clarke, Mr. F. J. de Soyres, and Mr. S. H. Swayne.

### SIXTH LIST OF BOOKS.

The figures in brackets refer to the figures after the names of the donors, and show by whom the volumes were presented. The books to which no such figures are attached have either been bought from the Library Fund or received through the *Journal.*

---

**Abercrombie, J.** *Pathological and Practical Researches on Diseases of the Brain and Spinal Cord* .. ... (1) 3rd Ed. 1834
**Adams, W.** ... *Subcutaneous Surgery* ... ... ... ... ... ... ... (1) 1857
**Adams, W.** ... *Reparative Process in Human Tendons* ... ... (1) 1860
*Aneurism, Observations on.* (Tr. and Ed. by J. E. Erichsen) ... (8) 1844
**Allaman, C.** ... *Des Alienés Criminels* ... ... ... ... ... ... ... (1) 1891
**Anstie, F. E.** ... *Neuralgia* ... ... ... ... ... ... ... ... ... ... (1) 1871

**Barlow, G. H.** ... *Manual of the Practice of Medicine* ... ... ... (1) 1856
**Beale, L.** ... ... *The Microscope in Clinical Medicine* ... ... ... (1) 1854
**Beissel, Dr.** (Ed.) *Aix-la-Chapelle.* (Tr. par Dr. Lermuseau) ... (1) 1891
**Bell, Sir C.** ... *The Hand* ... ... ... ... ... ... ... (1) 4th Ed. 1837
**Bell, John** .. *The Principles of Surgery.* 3 vols. in 4 ... ... (1) 1801-8
**Bell, Joseph** ... *Manual of Surgical Operations* ... ... ... 7th Ed. 1892
**Bell, T.** ... ... *The Anatomy, Physiology, and Diseases of the Teeth* (1) 2nd Ed. 1835
**Bennett, J. H.** ... *Introduction to Clinical Medicine* .. ... (1) 2nd Ed. 1853
**Bigg, H.** ... ... *Manual of Orthopœdy.* Part I.—*The Head and Neck* 1892
**Billing, A.** ... *First Principles of Medicine* ... ... .. (1) 5th Ed. 1849
**Bramwell, B.** ... *Atlas of Clinical Medicine.* ... ... Vol. II., Pt. I. 1892
**Brodie, C. G.** ... *Dissections Illustrated* ... ... ... ... ... Part I. [1892]
**Buck, A. H.** (Ed.) *Hygiene and Public Health.* 2 vols. ... ... ... (1) 1879
**Buren and E. L. Keyes, W. H.** *On the Genito-Urinary Organs* ... (1) 1875
**Burnett, W.** ... *Mediterranean Fever* ... ... ... ... ... ... ... (1) 1814

**Carmichael, J.** ... *Diseases in Children* ... ... ... ... ... ... ... ... 1892
**Carpenter, W. B.** *Principles of Human Physiology* ... ... (1) 4th Ed. 1853
**Cathell, D. W.** ... *Book on the Physician Himself* ... ... ...10th Ed. 1892
**Celsus, A. C.** ... *Medicinæ Libri Octo.* (Ed. by E. Milligan) (1) 2nd Ed. 1831
**Cheyne, W. W.** (Ed.) *Micro-Parasites in Disease* ... ... ... ... (1) 1886
**Chomel, A. F.** ... *Eléments de Pathologie Générale* ... ... (1) 4th Ed. 1856
**Christison, R.** ... *A Treatise on Poisons* ... ... ... ... (1) 3rd Ed. 1836
**Clarke, E.** ... *Eyestrain* ... ... ... ... ... ... ... ... ... ... 1892
**Cobbold, T. S.** ... *Parasites* ... ... ... ... ... ... ... ... ... (2) 1879
**Copeland, T.** ... *Observations on the Principal Diseases of the Rectum and Anus* ... ... .. ... ... ... (1) 2nd Ed. 1814
**Coupland, S.** ... *Notes on the Clinical Examination of the Blood and Excreta* ... ... ... ... ... ... ... 3rd Ed. 1892

**Courtenay, F. B.** *On the Cure of Stricture of the Urethra* ... ... (1) 1851
**Cunningham, D. J.** *Guide de Dissection.* (Adaptation par P. Kuborn) (1) 1890
**Currie, J.** ... ... *Medical Reports on the Effects of Water, etc.* (1) 2nd Ed. 1798

**Daniel, W. F.** ... *Topography and Diseases of Guinea* ... ... ... (1) 1849
*Diphtheria, Memoirs on.* (Ed. by R. H. Semple) ... ... ... ... (8) 1859
**Dixey, F. A.** ... *Epidemic Influenza* ... ... ... ... ... ... ... 1892
**Duncan, A.** ... *Prevention of Diseases in Tropical Campaigns* ... (1) 1888
**Dupuytren, Baron** *Leçons Orales de Clinique Chirurgicale.* (1) Tomes I. II. 1832-33
,, *Lesions of the Vascular System.* (Tr. and Ed. by F. le G. Clark) ... ... ... ... ... ... ... (8) 1854

**Ewald, C. A.** ... *Lectures on Diseases of the Digestive Organs.* (Tr. by R. Saundby) ... ... ... ... ... (1) Vol. II. 1892

**Fenwick, E. H.** *Golden Rules of Surgical Practice* ... ... 3rd Ed. [1892]
**Feuchtersleben, Baron** *Medical Psychology.* (Tr. by H. E. Lloyd and Ed. by B. G. Babington) ... ... ... ... (8) 1847
**Ford, E.** ... ... *Observations on the Disease of the Hip Joint, etc.* (1) 1794
**Fuller, H. W.** ... *Diseases of the Chest* ... ... ... ... ... ... ... (1) 1862

**Giles, G. M.** ... *Kála-azár and Beri-beri* ... ... ... ... ... ... (1) 1890
**Glover, R. M.** ... *Pathology and Treatment of Scrofula* ... ... ... (1) 1846
**Graves, E. J.** ... *A System of Clinical Medicine* ... ... ... ... (1) 1843
,, *Clinical Lectures on the Practice of Medicine.* 2 vols. (1) 2nd Ed. 1848
**Griffin, W. and D.** *Functional Affections of the Spinal Cord* .. ... (1) 1834
**Guy, W. A.** ... *Principles of Forensic Medicine.* 3 parts ... ...(1) 1843-44

**Harknett, J. J.** *Antiseptic Dry-air Treatment of Consumption.* 2nd Ed. 1892
**Harrison, R.** ... *The Dublin Dissector* ... ... ... ... (1) 5th Ed. 1838
**Harrison, R.** ... *The Surgical Disorders of the Urinary Organs.* (1) 2nd Ed. 1880
,, *The Prevention of Stricture and of Prostatic Obstruction* ... ... ... ... ... ... ... ... ... (1) 1881
**Henoch, E.** ... *Lectures on Children's Diseases.* 4th Ed. (Tr. by J. Thomson) ... ... .. ... ... (1) Vol. II. 1889
**"Historicus"** ... *Cocoa: All About It* ... ... ... ... ... ... ... ... 1892
**Holden, L.** ... *Human Osteology* ... ... .. ... ... (1) 4th Ed. 1869
**Hope, J.** ... ... *On the Diseases of the Heart* ... ... (8) 3rd Ed. 1839
*Hospitals, The Modern Practice of the London* ... .. ... ... ... (1)
**Husband, H. A.** *The Student's Handbook of the Practice of Medicine* (1) 2nd Ed. 1878
**Huxham, J.** ... *An Essay on Fevers* ... ... ... ... ... ... ... (1) [N.D.]

**Jackson, J.** ... *Letters to a Young Physician* ... ... (1) 4th Ed. 1856
**Jones, H.** ... ... *A Guide to the Examinations in Sanitary Science* ... 1892

**Jones, T. W.** ... *Ophthalmic Medicine and Surgery* ... ... ... (1) 1847

**Keating, J. M.** *Pronouncing Dictionary of Medical Terms* ... ... ... 1892
**Keyes, W. H. Buren and E. L.** *On the Genito-Urinary Organs* ... (1) 1875
**Kingzett, C. T.** *Nature's Hygiene* ... ... ... ... ... (1) 2nd Ed. 1884
**Kirchner, W.** ... *Handbuch der Ohrenheilkunde* ... ... (1) 3rd Ed. 1890
**Koecker, L.** ... *Diseases of the Jaws* ... ... ... ... ... ... (1) 1847

**Laennec, R. T. H.** *Treatise on Diseases of the Chest.* (Tr. by J. Forbes) (1) 4th Ed. 1834
**Lallemand, F.**... *A Treatise on Spermatorrhœa.* (Tr. and Ed. by H. J. McDougall) ... ... ... ... ... ... ... (1) 1847
**Lefebvre, A.** ... *Des Déformations Ostéo-Articulaires* ... ... ... (1) 1891
**Lescher, F. H.**... *Recent Materia Medica* ... ... ... (1) 3rd Ed. 1888
**Levillain, L.** ... *La Neurasthénie* ... ... ... ... ... ... ... (1) 1891
**Lonsdale, E. F.** *A Practical Treatise on Fractures* ... ... ... (1) 1838
,, *Lateral Curvature of the Spine* ... ... ... ... (1) 1847
**Lyons, R. D.** ... *Handbook of Hospital Practice* ... ... ... ... (1) [1858]

**Mackintosh, J.** *Principles of Pathology and Practice of Physic.* 2 vols. (1) 3rd Ed. 1832
**Mapother, E. D.** *Physiology and Disease* ... ... ... ... ... (1) 1864
**Marsden, A.** ... *Cancer* ... ... ... ... ... ... ... (8) 2nd Ed. 1874
**Mayo, H.** ... ... *Outlines of Human Physiology* ... ... ... ... (1) 1827
,, *Outlines of Human Pathology.* 2 parts ... (1) 1835–36
**Meadows, A.** ... *The Prescriber's Companion* ... ... (1) 3rd Ed. 1874
*Mechanical Exercise a Means of Cure* ... ... ... ... ... ... ... (8) 1883
**Milton, J. L.** ... *On the Treatment of Lupus* ... ... ... ... ... (1) 1866
**Monro, A.** ... *The Anatomy of the Human Bones, Nerves, and Lacteal Sac and Duct* ... ... ... ... ... (1) 1788
**Moullin, C. W. M.** *The Operative Treatment of Enlargement of the Prostate* 1892
**Moure, E. J.** ... I. *Abcès du Larynx dans la Scarlatine.* II. *Un Nouveau Cas de Chancre Induré de la Fosse Nasale* ... ... 1892
**Müller, J.** ... *Elements of Physiology* (1) Parts II., III., IV., 1838; (1) V., 1839

**Newman, D.** ... *Malignant Disease of the Throat and Nose*... ... ... 1892
**Niemeyer, F. v.** *Text-book of Practical Medicine.* 8th Ed. (Tr. by. G. H. Humphreys and C. E. Hackley.) 2 vols. (1) 1880

**Oesterlen, F.** ... *Medical Logic.* (Tr. and Ed. by G. Whitley) ... (8) 1855
"*Oesypum*" *to* "*Lanoline,*" *From* ... ... ... ... ... ... ... ... 1892
**Oven, B. v.** ... *On the Decline of Life* ... ... ... ... ... (1) 1853

**Parkes, L. C.** ... *Hygiene* ... ... ... ... ... ... ... ... 3rd Ed. 1892
**Pereira, J.** ... *Materia Medica* (1) Vol. I., 3rd Ed. 1849; (1) Vol. II. Part I., 3rd Ed. 1850, Part II., 4th Ed. 1857
**Pettigrew, J. B.** *Physiology of the Circulation* ... ... ... ... (1) 1874
**Rayer, P.**... ... *Diseases of the Skin.* 2nd Ed. (Tr. by R. Willis) (1) 1835

| | | | |
|---|---|---|---|
| **Reynolds, J. R.** | *Epilepsy* | (1) | 1861 |
| **Richardson, B. W.** | *The Coagulation of the Blood* | (1) | 1858 |
| **Ringer, S.** | *A Handbook of Therapeutics* | (4) 2nd Ed. | 1891 |
| **Roberts, R. L.** | *Nursing and Hygiene* | (1) | 1890 |
| **Robins, E. C.** | *Modern Hospital Construction* | (1) | 1883 |
| **Roger, H.** | *Séméiotique des Maladies de l'Enfance* | (1) | 1864 |
| **Rowland, R.** | *A Treatise on Neuralgia* | (1) | 1838 |
| **Rudolphi, K. A.** | *Grundriss der Physiologie.* 2 vols. in 1 | (1) | 1821–28 |
| **Sansom, A. E.** | *The Diagnosis of Diseases of the Heart and Thoracic Aorta* | | 1892 |
| **Savill, T. D.** | *Epidemic Skin Disease* | | 1892 |
| **Schoenlein, J. L.** | *Klinische Vorträge in dem Charité-Krankenhause zu Berlin* | (1) 3rd Ed. | 1843 |
| **Sebillote, R.** | *Intoxications per le sublimé corrosif chez les femmes en couches* | (1) | 1891 |
| **Semmola, M.** | *Die Alte und die Neue Medizin.* (Ueber. von V. Meyer) | (1) | 1885 |
| **Sharpe, S.** | *A Treatise on the Operations of Surgery* | (1) | 1788 |
| **Smith, F. A. A.** | *Keep Your Mouth Shut* | | 1892 |
| **Smith, H.** | *Stricture of the Urethra* | (1) | 1857 |
| **Spender, J. K.** | *On Ulcers* | (1) | 1868 |
| **Spittal, R.** | *A Treatise on Auscultation* | (1) | 1830 |
| **Stacpoole, Florence** | *Advice to Women on the Care of the Health before, during, and after Confinement* | | 1892 |
| ,, ,, | *Our Sick, and How to Take Care of Them* | | 1892 |
| **Stewart, T. G.** | *Lectures on the Nervous System* | (1) | 1884 |
| **Swanzy, H. R.** | *Diseases of the Eye* | 4th Ed. | 1892 |
| **Thomas, H. O.** | *Fractures, &c. of the Lower Extremities* | (1) | 1890 |
| **Thompson, Sir H.** | *Stricture of the Urethra* | (1) 3rd Ed. | 1869 |
| ,, | *Diseases of the Urinary Organs* | (1) 4th Ed. | 1876 |
| **Thompson, R. E.** | *Physical Examination of the Chest* | (1) | 1879 |
| **Tommasi-Crudeli, Prof.** | *Rome and Malaria* | | 1892 |
| **Tuckey, C. L.** | *The Value of Hypnotism in Chronic Alcoholism* | | 1892 |
| **Tuke, D. H.** | *Reform in Treatment of the Insane* | | 1892 |
| **Tuson, E. W.** | *On Curvature of the Spine* | (1) | 1841 |
| **Tyson, J.** | *Practical Examination of Urine* | (1) 4th Ed. | 1883 |
| **Vogel, J.** | *Disorders of the Blood.* (Tr. and Ed. by C. C. Day) | (1) | 1856 |
| **Wade, R.** | *Stricture of the Urethra* | (1) | 1853 |
| **Walley, T.** | *Meat Inspection* | (1) | 1890 |
| **Wethered, F. J.** | *Medical Microscopy* | (9) | 1892 |
| **Whately, T.** | *Observations on Ulcers, &c.* | (1) 2nd Ed. | 1816 |
| **Wilks, S.** | *Pathological Anatomy* | (1) | 1859 |
| **Wilson, W. J. E.** | *Practical and Surgical Anatomy* | (1) | 1838 |

### TRANSACTIONS, REPORTS, JOURNALS, &c.

*The List of current Periodicals to be seen in the Library is given in the* Journal *for March.*

American Gynecological Society, Transactions of the (1) Vol. XVI. 1891
American Journal of Ophthalmology, The ... ... ... Vol. VIII. 1891
Australasian Medical Directory ... ... ... ... ... ... ... ... ... 1892
Bibliotheca Medico-Chirurgica ... ... ... ... ... ... ... ... 1892
Calendar of the Royal College of Surgeons in England ... ... ... 1892
China Medical Missionary Journal, The (3) Vol. IV., 1890; Vol. V. 1891
Dublin Journal of Medical Science, The (1) Vols. LIII.—LXVII. 1872–79
Epidemiological Society of London, Transactions of the
N. S. (1) Vols. I., II., 1883; (1) IV.—VII. 1885–89
Hospitals—
Bristol Royal Infirmary Reports ... ... ... (7) Vol. I. 1878–79
St. Thomas's Hospital Reports. N. S. (5) Vols. I.—XVIII. 1870–90
Jahrbücher, Schmidt's (1) Bd. 97—104, 1858–59; (1) Bd. 109—112, 1861; (1) Bd. 115—122, 1862-64; (1) Bd. 127—130 1865–66
Liverpool Medico-Chirurgical Journal, The
(6) Vols. VIII., IX., 1888-89; Vol. XI. 1891
London Medical Review, The ... ... ... ... ... (1) Vol. I. 1808
London, Transactions of the Pathological Society of. (1) Vols. IV.—XV., 1853-64; (1) Index, Vols. I.—XV., 1864; (1) Vols. XVI.—XXXVII., 1865-86; (1) Index, Vols. XVI.—XXXVII., 1887; (1) Vols. XXXVIII.—XLI. ... ... ... .. ... ... 1887–90
Manchester, Transactions of the Pathological Society of. Vol. I. 1891–92
Marine-Hospital Service of the United States, Annual Reports of the Supervising Surgeon-General of the ... ... ... ... (1) 1890–91
Medical Annual, The ... ... ... ... ... ... ... ... ... ... (8) 1891
Medical Review ... ... ... ... ... ... ... ... Vol. XXV. 1892
Navy, Statistical Report on the Health of the, 1890 .. ... (1) 1891
Ohio, Fifth Annual Report of the State Board of Health of the State of ... ... ... ... ... ... ... ... ... ... ... ... (1) 1891
Practitioner, The ... ... ... ... ... ... ... ... (1) Vols. I.—IV. 1868–70
Progrès médical, Le ... ... ... ... ... 2e Ser. Tome XV. 1892
Registrar-General of Births, Deaths, and Marriages in England, Reports of... ... ... ... ... ... ... ... ... ... ... ... (1) 1887–91
Retrospect of Medicine, Braithwaite's. (8) Vols. LXXV.-LXXVII. 1877–78
Sanitarian, The (1) Vols. XVIII.-XX., 1887-88; (1) XXII., XXIII. 1889
Sanitary Institute of Great Britain, Transactions of the
(1) Vols. I.—XI. 1880–91
Surgeons in London, List of the Members of the Royal College of (1) 1825–1827
Surgeons of England, List of the Fellows and Members of the Royal College of ... ... ... ... ... ... ... ... ... ... (1) 1844
Year-book of Treatment, The ... ... ... ... ... ... ... ... (1) 1884
Zeitschrift der k. k. Gesellschaft der Arzte in Wien ... ... (1) 1862–63

LIST OF SUBSCRIBERS

TO

### The Bristol Medico-Chirurgical Journal.

*DECEMBER*, 1892.

---

### England.

### BEDFORDSHIRE.

BEDFORD.
W. G. Nash

### CHESHIRE.

CHESTER.
R. E. Burges

EGREMONT.

T. W. A. Napier

MACCLESFIELD.
W. R. Etches

### CORNWALL.

BODMIN.
W. Pearse

NEW QUAY.
A. Hardwick

PADSTOW.
H. F. Marley

PENZANCE.
W. S. Bennett
J. A. Fox
J. Symons

POLRUAN.
W. H. Torbock

ST. AUSTELL.
W. Mason

ST. JUST.
R. B. Searle

TRURO.
E. Rundle

### DEVONSHIRE.

BABBACOMBE.
G. F. P. Pizey

BAMPTON.
T. A. Guinness

BARNSTAPLE.
T. Clark
C. H. Gamble
J. Harper
W. A. G. Laing
F. Penny

BRIXHAM.
G. C. Searle

BROADCLYST.
J. Somer

BUDLEIGH SALTERTON.
H. F. Semple

CHAGFORD.
A. D. Hunt

CHUDLEIGH.
C. H. Wade

DARTMOUTH.
J. H. Harris

DAWLISH.
A. de W. Baker

DEVONPORT.
J. Harrison
F. E. Row

EXETER.
J. M. Ackland
A. G. Blomfield
H. Davy
P. M. Deas
E. J. Domville
W. Gordon
A. W. Kempe
E. B. Steele-Perkins
L. H. Tosswill

HONITON.
T. W. Shortridge

NEWTON ABBOT.
E. Haydon

OTTERY ST. MARY.
F. A. Gray

### DEVONSHIRE *(Continued)*.

PAIGNTON.

J. Alexander

PLYMOUTH.

R. H. Clay
E. L. Fox
R. H. Lucy
E. B. Thomson
W. L. Woollcombe

PLYMPTON.

W. D. Stamp

ST. MARY CHURCH.

T. Finch

SALCOMBE.

A. H. Twining

SOUTH MOLTON.

E. Furse

TAVISTOCK.

M. T. Leamon

TORQUAY.

W. Odell
R. Pollard
W. Powell

UFFCULME.

F. Morgan

WINKLEIGH.

J. H. Norman

YELVERTON.

A. P. Prowse

### DORSETSHIRE.

BEAMINSTER.

T. P. Daniel
F. P. Kitson

DORCHESTER.

F. B. Fisher
P. W. Macdonald

PARKSTONE.

W. H. Masters

POOLE.

A. C. Kemble
J. McNicoll

PORTLAND.

G. H. Lilley

STURMINSTER NEWTON.

J. C. Leach

WEYMOUTH.

R. W. Carter
J. M. Lawrie

WIMBORNE.

A. J. H. Crespi

### ESSEX.

NEWPORT.

W. A. Smith

### GLOUCESTERSHIRE.

BRISTOL.

W. R. Ackland
G. Adams
J. B. Adams
G. F. Atchley
W. M. Barclay
T. G. L. Baretti
B. J. Baron
A. E. Blacker
E Blacker
A. F. Blagg
E. C. Board
J. G. Boyce
J. Broom
E. F. H. Burroughs
J. P. Bush
J. Cameron
A. Carr
W. F. Carter
J. M. Clarke
E. W. Coathupe
R. W. Coe
T. V. Coker
T. J. Colman
H. Cook
G. G. Corbould
W. Cotton
F. R. Cross
J. Dacre
D. S. Davies
H. F. Devis
H. R. Dew
N. C. Dobson
W. Dowson
E. L. W. Dunbar
F. H. Edgeworth
W. Elder
C. Elliott
J. Ewens
R. G. Fendick
E. L. Fox
W. J. Fyffe
A. N. G. Gibbs
J. Gill
G. A. Gloag
W. C. Goodden
W. G. Grace
J. S. Griffiths
L. M. Griffiths
C. D. G. Hailes
A. H. Haines
A. J. Harrison
W. H. Harsant
A. G. Hayman
J. C. Heaven
C. K. C. Herapath
H. E. Hetling
H. Hill
F. F. Hills
A. W. W. Hoffman
General Hospital
G. A. Imlay
Royal Infirmary
J. Irving
D. G. Johnston
F. P. Lansdown
R. G. P. Lansdown
A. E. A. Lawrence
H. Lawrence
E. L. Lees
Museum and Library
W. E. Lloyd
F. T. B. Logan
H. W. G. Macleod
W. T. Maddison
H. Marshall
C. E. Matthews
T. O. Mayor
J. S. Metford
J. Milne
H. F. Mole
C. A. Morton
G. T. Myles
B. G. Neale
W. N. Nevill
C. Newman
W. H. C. Newnham
J. Newstead
T. D. Nicholson
J. A. Norton
A. Ogilvy
G. S. Page
G. Parker
J. H. Parry
G. C. P. Pauli
W. J. Penny
C. F. Pickering
W. E. Pountney
J. J. Powell
A. Prichard
A. W. Prichard
J. E. Prichard
A. Pring

### GLOUCESTERSHIRE *(Continued).*

BRISTOL *(continued).*

A. B. Prowse
B. M. H. Rogers
W. B. Roué
C. K. Rudge
H. T. Rudge
J. E. Shaw
E. J. Sheppard
W. E. Shorland
D. Sinclair
E. M. Skerritt
G. M. Smith
J. G. Smith
R. Smith
R. S. Smith
S. Smith
R. V. Solly
A. A. Sproule
G. M. Stansfeld
C. Steele
W. H. Stevens
F. W. Stoddart
J. Swain
J. G. Swayne
S. H. Swayne
W. C. Swayne
J. Taylor
W. J. Tivy
H. Waldo
C. H. Walker
C. H. Wallace
J. H. Wathen
T. Webster
J. Wilding
P. W. Williams
H. W. Windsor-Aubrey
C. Wintle

CHELTENHAM.

Cheltenham Library
F. A. A. Smith
L. Winterbotham

CHIPPING SODBURY.

A. Grace

CINDERFORD.

W. C. Heane

CIRENCESTER.

W. R. Cossham

COLEFORD.

L. B. Trotter

DOWNEND.

H. Skelton

FISHPONDS.

W. Brown

GLOUCESTER.

R. W. Batten
T. Edis
T. S. Ellis
J. G. Soutar

HAMBROOK.

E. Crossman
W. F. B. Eadon

HAWKESBURY-UPTON.

W. I. Cox

KINGSWOOD.

H. Grace

MORETON-IN-MARSH.

L. K. Yelf

STAPLE HILL.

W. Murray

STAPLETON.

H. T. S. Aveline
H. A. Benham

STOKE BISHOP.

C. E. Wedmore

STONEHOUSE.

G. T. B. Watters

STOW-ON-THE-WOLD.

E. Dening

STROUD.

A. S. Cooke
E. A. Howsin
F. W. Storry

TETBURY.

W. Wickham

THORNBURY.

E. M. Grace
T. H. Taylor
L. H. Williams

WESTBURY-ON-TRYM.

W. Duncan
H. Ormerod

WINTERBOURNE.

R. Eager

WOTTON-UNDER-EDGE.

D. H. Forty

### HAMPSHIRE.

BOURNEMOUTH.

D. C. Embleton
J. Horsfall

CARISBROOKE.

J. Groves.

LYMINGTON.

J. Rendall

ST. MARY BOURNE.

A. W. Riddell

SOUTHSEA.

J. W. Cousins

VENTNOR.

R. Robertson

### HEREFORDSHIRE.

HEREFORD.

W. C. Whitfeld

### HERTFORDSHIRE.

ST. ALBANS.

A. H. Boys

### KENT.

TUNBRIDGE WELLS.

C. Wilson

### LANCASHIRE.

LIVERPOOL.

G. A. Hawkins-Ambler
Medical Institution

POULTON-LE-FYLDE.

P. H. Day

### MIDDLESEX.

LONDON.

J. Abercombie
A. E. Barker
J. Berry
L. Browne
J. G. Davey
J. F. Evans
A. P. Gould
J. M. Granville
G. W. Grote
M. Handfield-Jones
W. P. Herringham
W. H. A. Jacobson
C. M. Jessop
H. M. Jones
J. Lister
E. J. Maclean
J. Macready
A. L. Marshall
W. Pippette
R. D. Powell
M. A. Ruffer
W. J. Seward
N. Smith
Royal Med. and Chir. Society
J. A. Watson
F. J. Wethered
G. S. Woodhead

TOTTENHAM.

W. H. Plaister

### MONMOUTHSHIRE.

ABERGAVENNY.

W. J. Clapp

RHYMNEY.

W. W. Jones
T. H. Redwood

NEWPORT.

W. Basset
C. B. Gratte
H. R. Hudson
T. Limbery
O. E. B. Marsh
A. G. Thomas
J. T. Thomas
H. E. Williams

RISCA.

G. B. Robathan

PONTYPOOL.

S. B. Mason

TREDEGAR.

G. A. Brown

### NORTHUMBERLAND.

BAMBURGH.

J. G. Macaskie

### NOTTINGHAMSHIRE.

NOTTINGHAM.

C. B. Taylor
W. H. Walter

### OXFORDSHIRE.

HENLEY-ON-THAMES.

J. Lidderdale

MIDDLETON CHENEY.

E. H. Openshaw

OXFORD.

R. W. Doyne
H. P. Symonds

### SOMERSETSHIRE.

BATH.

G. A. Bannatyne
G. E. Bloxam
A. B. Brabazon
T. Cole
F. S. Cowan
S. Craddock
W. M. Ellis
A. E. W. Fox
H. W. Freeman
H. F. A. Goodridge
T. B. Goss
F. P. Hoblyn
H. C. Hopkins
L. J. King
F. Lace
H. Lane
T. P. Lowe
R. J. H. Scott
E. Skeate
J. K. Spender
L. A. Weatherly

BECKINGTON.

W. G. Evans

BRIDGWATER.

T. L. Laxton
F. J. C. Parsons
J. B. Sincock

BRISLINGTON.

B. B. Fox
C. H. Fox

BURNHAM.

A. W. C. Peskett

CHEDDAR.

R. W. Statham

CHEW MAGNA.

E. T. Hale

CLEVEDON.

T. Davis
J. A. F. Sawyer
S. Skinner

CREWKERNE.

E. J. Cave
W. W. Webber

### SOMERSETSHIRE *(Continued)*.

DULVERTON.

G. F. Sydenham

FRESHFORD.
C. E. S. Flemming

FROME.
E. Cockey
F. Parsons
J. M. Rattray

GLASTONBURY.
J. A. Bright

HIGHBRIDGE.
R. Wade

ILMINSTER.
C. Munden

KEYNSHAM.
G. G. D. Willett

LANGPORT.
J. Morgan

MIDSOMER NORTON.
A. Waugh
A. H. Whicher

MILVERTON.
C. Randolph

NORTH PETHERTON.
C. F. Hawkins

PILL.
R. A. Ross

PORTISHEAD.
W. Monckton
C. A. Wigan

SHEPTON MALLET.
J. T. Hyatt

TAUNTON.
H. Alford
H. J. Alford
Taunton Hospital
W. Liddon

TEMPLE CLOUD.
T. Martin

TWERTON-ON-AVON.
J. Wigmore

WEDMORE.
J. Ford
R. P. Tyley

WELLINGTON.
J. Meredith
T. E. P. Prideaux

WELLS.

F. J. B. Bateman

W. Fairbanks

A. L. Wade

WESTON-SUPER-MARE.
G. E. Alford
C. P. Crouch
G. B. Fraser
T. C. Grey
E. F. Martin
W. H. G. Phelps
G. F. Rossiter
R. Roxburgh

WORLE.
F. St. J. Kemm

WRINGTON.
A. H. Benson
H. W. Collins

YEOVIL.
P. S. H. Colmer
E. Vernon

### STAFFORDSHIRE.

TAFFORD.
J. Scott

### SURREY.

EPSOM.
R. Davis

ESHER.
J. B. Fry

WOKING.
F. C. Palmer

### SUSSEX.

BRIGHTON.
H. H. Tomkins

ST. LEONARD'S-ON-SEA.
W. H. Spencer

### WARWICKSHIRE.

BIRMINGHAM.
L. Tait

### WILTSHIRE.

BRADFORD-ON-AVON.

W. J. A. Adye

J. Beddoe

BURBAGE.
A. E. N. Browne

CALNE.
W. S. Batten

DEVIZES.
C. Eddowes
A. M. Gray

FOVANT.
C. Clay

MALMESBURY.
R. Kinneir

SALISBURY.
L. S. Luckham
H. J. Manning

SWINDON.

E. D. Duffett

H. W. M. Hayes

W. Howse

J. C. Maclean

WARMINSTER.
J. Hinton
R. L. Willcox

### WORCESTERSHIRE

POWICK.
R. H. Norgate

### YORKSHIRE.

HARROGATE.
A. O. Jones

HULL.
J. B. Close

SHEFFIELD.
S. Snell

### Ireland.

### ANTRIM.

BELFAST.

J. W. Browne

### Scotland.

### EDINBURGHSHIRE.

EDINBURGH.

J. W. Ballantyne
Y. J. Pentland

### Wales.

### BRECONSHIRE.

BRECON.
A. Whyte

BRYNMAWR.
G. H. Browne

SENNY BRIDGE.
W. R. Jones

TALGARTH.
W. Howells

### CARDIGANSHIRE.

NEW QUAY.
E. R. Jones

### CARMARTHENSHIRE.

CARMARTHEN.
G. J. Hearder

FERRYSIDE.
P. Williams

LLANELLY.
D. J. Williams

### GLAMORGANSHIRE.

ABERAMAN.
J. B. James

ABERDARE.
E. Jones

CAERPHILLY.
J. Lewellyn

CARDIFF.

W. T. Edwards

J. Milward

D. R. Paterson

W. Price

R. Prichard

A. Sheen

Medical Society

J. T. Thompson

H. R. Vachell

C. Williams

DOWLAIS.
E. H. Jones

LLANDAFF.

J. Arthur

MERTHYR TYDVIL.
J. L. W. Ward

PENARTH.
W. A. Moynan

PORTH.
H. N. Davies

SWANSEA.

W. F. Brook

E. Davies

F. Knight

G. Padley

TREHARRIS.

W. W. Leigh

### PEMBROKESHIRE.

PENALLY.
B. Kendall

### Channel Islands.

GUERNSEY.
N. Webster

### Belgium.

COURTRAI.
Dr. Lauwers

### Greece.

CORFU.
Dr. Mavrojanni

### Italy.

NAPLES.
C. Scimonelli

### Russia.

ODESSA.
G. Schleicher

### Switzerland.

DAVOS-PLATZ.
W. R. Huggard

### Algeria.

ALGIERS.
W. Thomson

### Cape Colony.

FORT BEAUFORT.
J. Shaw

### United States.

NEW YORK.
J. B. Jackson

### Victoria.

BRADFORD.
G. H. Skinner

### INDEX.

Abortion, 120.
Alvarenga prize, 69.
American surgical association, Transactions of (*Review*), 317.
Anæsthetics—H. Davis (*Review*), 318.
Anatomy, Practical—H. C. Boenning (*Review*), 134.
Annual of the universal medical sciences (*Review*), 318.
Antiseptics in midwifery, 118.
Antrum, Diseases of, 123.
Asphyxia in the new-born infant, 121.
Atlas of clinical medicine—Dr. B. Bramwell (*Review*), 135, 213.
Auditory centre, The, 44.
Auld, Dr. A. G.—The pathological histology of bronchial affections, pneumonia, and fibroid pneumonia (*Review*), 136
Aural speculum, Dr. Ward Cousins's, 43.

Bacteriological diagnosis — Dr. J. Eisenberg (*Review*), 221.
Bacteriology and practical medicine, 292.
Bacteriology, Essentials of—Dr. M. V. Ball (*Review*), 218.
Ballance and W. Edmunds, C. A.—A treatise on the ligation of the great arteries in continuity (*Review*), 311.
Ball, Dr. M. V.—Essentials of bacteriology (*Review*), 218.
Barclay, W. M.-Report on surgery, 31
Baron, Dr. B. J.—Report on laryngology, 123.
Barr, Dr. J.—Treatment of typhoid fever (*Review*), 214.
Bath as a health-resort—Dr. A. B. Brabazon, 254.
Blomfield, Dr. A. G.—Case of locomotor ataxy with Charcot's joint disease, 249.
Boenning, H. C.—A treatise on practical anatomy (*Review*), 134).
Bourke, Capt. J. G. — Scatalogic rites (*Review*), 56.
Brabazon, Dr. A. B.—Bath as a health-resort, 254.
Bramwell, Dr. B.—Atlas of clinical medicine (*Review*), 135, 213.
Bristol Medical School—A. Prichard, 264.
Bristol Medico-Chirurgical Society, 59, 144, 329.
Bronchial affections, etc., Pathological histology of—A. G. Auld (*Review*), 135.
Brown, Dr. J.—Colour test chart (*Review*), 48.
Buchanan, Sir G., 149.
Burder, Dr. G. F., 67.
Buret, Dr. F.—Syphilis in ancient and prehistoric times (*Review*), 220.

Cancer in women—Dr. H. Snow (*Review*), 128.
Charcot's joint disease, Locomotor ataxy with.—Drs. H. Davy and A. G. Blomfield, 249.
Chemistry, Practical—Dr. S. Rideal (*Review*), 139.
Children, Diseases of—P. E. Muskett (*Review*), 140.
Chinese, The—Dr. R. Coltman (*Review*), 323.
Cholera, 292.
Circumcision, History of—Dr. P. C. Remondino (*Review*), 141.
Clarke, Dr. J. M.—Report on medicine, 107.
Clinical charts (*Review*), 54.
Colour test chart—Dr. J. Brown (*Review*), 48.
Coltman, Dr. R.—The Chinese (*Review*), 323.
Consumption—Dr. N. S. Davis (*Review*), 315.
Coombe, R.—Epitome of British Pharmacopœia (*Review*), 50.
Coroner, The office of — F. W. Lowndes (*Review*), 139.
Coupland, Dr. S.—Examination of sputum, &c. (*Review*), 49.
Cross, F. R.—Reports on ophthalmology, 36, 208.
Curgenven, J. B. — The disinfection of scarlet fever and other infectious diseases by antiseptic inunction (*Review*), 316.

Davis, Dr. N. S.—Consumption (*Review*), 315.
Davis, H.—Anæsthetics (*Review*), 318.
Davy, Dr. H.—Case of locomotor ataxy with Charcot's joint disease, 249.
Diphtheria, 201, 295.
Disinfection of scarlet fever—J. B. Curgenven (*Review*), 316.
Domestic animals, Age of the—Dr. R. S. Huidekoper (*Review*), 219
Doudney, Dr. G. H.—The water cure in the bedroom (*Review*), 134.

Ear disease, Cerebral abscess following, 42.
Ear, Diseases of the—Dr. U. Pritchard (*Review*), 48.
Ear, The human—Dr. A. Politzer (*Review*), 135.
Early life, Palsies of, 107.
Edmunds, C. A. Ballance and W.—A treatise on the ligation of the great arteries in continuity (*Review*), 311.
Eisenberg, Dr. J.—Bacteriological diagnosis (*Review*), 221.
Electricity, Medical—Drs. D. D. Stewart and E. S. Lawrance (*Review*), 317.
Epilepsy, 110.
Epistaxis, 125.
Examination of sputum, &c.—Dr. S. Coupland (*Review*), 49.
Eye symptoms in acromegaly, 39; in locomotor ataxy, 40.
Eye therapeutics, 41, 42.

Failure, The teachings of—Dr. E. M. Skerritt, 229.
Fevers—Dr. J. W. Moore (*Review*), 320.
Forceps delivery during the last fifty years—Dr. J. G. Swayne, 153.
Fyffe, Dr. W. J.—A recent outbreak of rötheln, 1.

Goitre, Strophanthus in exophthalmic, 41.
Gotch and V. Horsley, F.—On the mammalian nervous system (*Review*), 213.
Griffiths, L. M.—Reports on obstetrics, 117, 303.
Guy's hospital reports (*Review*), 319.
Gymnastics, Educational—H. Nissen (*Review*), 133.
Gynæcology, Congress of, 68.

Hæmoptysis, Treatment of, 199.
Hare-lip—W. Rose (*Review*), 130.
Harsant, W. H.—Reports on otology, 42, 309; report on surgery, 113.
Hartridge, G.—The ophthalmoscope (*Review*), 216.
Haydon, F. — Ophthalmic charts (*Review*), 51.
Hay-fever.—Dr. P. W. Williams, 84.
Herman, Dr. G. E.—First lines in midwifery (*Review*), 140.
Horsley, F. Gotch and V.—On the mammalian nervous system (*Review*), 213.
Huidekoper, Dr. R. S.—Age of the domestic animals (*Review*), 219.
Hypnotic suggestion—Dr. G. Kingsbury (*Review*), 132.
Hysterectomy, 301.

Inductive method in medicine—Dr. W. Murray (*Review*), 315.
Infectious diseases in schools (*Review*), 56.
Influenza, 296; Dr. R. Sisley (*Review*), 54.
Intestinal surgery, 113.
International clinics (*Review*), 55, 215.
Iodoform in tubercular lesions, 34.
Ireland, Transactions of the Royal Academy of Medicine in (*Review*), 139.

Jefferson Medical College of Philadelphia, 150.
Jessop, C. M.—Saturn's kingdom (*Review*), 51.
Johns Hopkins hospital reports (*Review*), 325.
Jones, Dr. H. M. — Diseases of women (*Review*), 49.

Kingsbury, Dr. G.—The practice of hypnotic suggestion (*Review*), 132.
Knee-jerk, The quantitative estimation of the—Dr. E. J. Maclean, 20.

Larkin, F. C.—Outlines of practical physiological chemistry (*Review*), 131.
Laryngology, Report on—Dr. B. J. Baron, 123.
Lawrance, Drs. D. D. Stewart and E. S. — Essentials of medical electricity (*Review*), 317.
Lawrence, Dr. A. E. A.—Extra-uterine pregnancy, 73.
Lawrie, Dr. J. Macpherson—A case of large ovarian tumour in a young girl, 186

Lead poisoning—Dr. T. Oliver (*Review*), 129.
Leeward Islands medical journal (*Review*), 52.
Leprosy—Dr. G. Thin (*Review*), 129.
Lewers, Dr. A. H. N.—A practical text-book of the diseases of women (*Review*), 219.
Library of the Bristol Medico-Chirurgical Society, 62, 146, 223, 332.
Library of the Surgeon-General's office, United States Army, Index-catalogue of (*Review*), 139.
Ligation of arteries—C. A. Ballance and W. Edmunds (*Review*), 311.
Lock, J. G.—Tenby as a health-resort, 97.
Lowndes, F. W.—Reasons why the office of coroner should be held by a member of the medical profession (*Review*), 139.

Maclean, Dr. E. J.—The quantitative estimation of the knee-jerk, 20.
McBride, Dr. P.—Diseases of the throat, nose, and ear (*Review*), 137.
Materia medica and therapeutics—Dr. J. V. Shoemaker (*Review*), 47.
Mediastinal tumours, The pathology of—Dr. J. L. Steven (*Review*), 220.
Medical annual (*Review*), 53.
Medical magazine (*Review*), 216.
Medical diary (*Review*), 337.
Medicine, Contributions to practical —Sir J. Sawyer (*Review*), 48.
Medicine, Principles and practice of —Dr. W. Osler (*Review*), 211.
Medicine, Reports on—Dr. J. M. Clarke, 107; Dr. J. E. Shaw, 292; Dr. E. M. Skerritt, 26; Dr. R. S. Smith, 201.
Middlesex hospital reports (*Review*), 133.
Midwifery, First lines in—Dr. G. E. Herman (*Review*), 140.
Midwives, 122.
Moore, Dr. J. W.—Text-book of the eruptive and continued fevers (*Review*), 320.
Morton, C. A.—A case of multiple symmetrical joint lesions, 189; a rare form of spina bifida, 14.
Multiple symmetrical joint lesions—C. A. Morton, 189.
Murray, Dr. W.—Illustrations of the inductive method in medicine (*Review*), 315.
Muskett, P. E.—Prescribing and treatment in the diseases of infants and children (*Review*), 140.

Nasal discharges, 124.
Nerve prostration—Dr. R. Roose (*Review*), 50.
Nervous system, Mammalian—F. Gotch and V. Horsley (*Review*), 213
Nervous system, Diseases of—Dr. J. A. Ormerod (*Review*), 217.
Neurologie, Archives de (*Review*), 316.
New York Academy of Medicine, Transactions of (*Review*), 131.
Nissen, H.—A B C of the Swedish system of educational gymnastics (*Review*), 133.

Obstetrics, Reports on—L. M. Griffiths, 117, 303.
Oliver, Dr. H.—Lead poisoning (*Review*), 129.
Ophthalmoscope, The—G. Hartridge (*Review*), 216.
Ophthalmic charts—F. Haydon (*Review*), 51.
Ophthalmology, Reports on—F. R. Cross, 36, 208.
Ormerod, Dr. J. A.—Diseases of the nervous system (*Review*), 129.
Osler, Dr. W.—The principles and practice of medicine (*Review*), 211.
Otology, Reports on—W. H. Harsant, 42, 309.
Ovarian tumour in a young girl, Large—Dr. J. Macpherson Lawrie, 186.
Oxygen in medicine, 26.

Page, H. W.—Railway injuries (*Review*), 127.
Pathology and bacteriology, Journal of (*Review*), 322.
Penny, W. J.—Report on surgery, 201.
Perineum, Support of, 120.
Pharmacopœia, Epitome of British —R. Coombe (*Review*), 150.
Phthisis, Treatment of, 198.
Physiological chemistry—F. C. Larkin (*Review*), 141.
Plymouth medical society, 331.
Pneumonia, Treatment of, 195, 296.
Politzer, Dr. A.—The anatomical and histological dissection of the human ear in the normal and diseased condition (*Review*), 135.
*Post-partum* hemorrhage, 121.
Pregnancy, Extra-uterine—Dr. A. E. A. Lawrence, 73.

Prescriber's companion — Dr. T. Savill (*Review*), 129.
Prichard, A. — Bristol Medical School, 264.
Pritchard, Dr. U.—Diseases of the ear (*Review*), 48.
Prostatectomy, 297.
Psycho-therapeutics — Dr. C. L. Tuckey (*Review*), 132.
Puerperal pyrexia, 119.
Pye, W.—Surgical handicraft (*Review*), 218.

Railway injuries—H. W. Page (*Review*), 127.
Remondino, Dr. P. C.—History of circumcision (*Review*), 141.
Rideal, Dr. S.—Practical chemistry for medical students (*Review*), 139.
Roose, Dr. R.—Nerve prostration (*Review*), 50.
Rose, W.—Hare-lip and cleft palate (*Review*), 130.
Rötheln, A recent outbreak of—Dr. W. J. Fyffe, 1.

Sarcoma, Uveal—36.
Saturn's kingdom—C. M. Jessop (*Review*), 57.
Savill, Dr. T.—Prescriber's companion (*Review*), 129.
Sawyer, Sir J.—Contributions to practical medicine (*Review*), 48.
Scatalogic rites—Capt. J. G. Bourke (*Review*), 56.
Scraps, 69, 150, 227, 339.
Shaw, Dr. J. E.—Report on medicine, 292.
Sheffield medical journal (*Review*), 321
Shoemaker, Dr. J. V.—Materia medica and therapeutics (*Review*), 47.
Sick, Preparations for the, 57, 142, 222, 327.
Sisley, Dr. R.—Influenza (*Review*), 54.
Skerritt, Dr. E. M.—Report on medicine, 26; the teachings of failure, 229.
Smith, N. — Spasmodic wry-neck (*Review*), 51.
Smith, Dr. R. S.—Report on medicine, 195.
Snow, Dr. H.—The proclivity of women to cancerous diseases and to certain benign tumours (*Review*), 128.
Spina bifida, A rare form of—C. A. Morton, 14.
Spinal surgery, 201.
St. Bartholomew's hospital reports (*Review*), 319.
Steven, Dr. J. L.—The pathology of mediastinal tumours (*Review*), 220.
Stewart and E. S. Lawrance, Drs. D. D. — Essentials of medical electricity (*Review*), 317.
St. Thomas's hospital reports (*Review*), 320.
Surgery, A manual of operative—F. Treves (*Review*), 145.
Surgery, Reports on—W. M. Barclay, 31; W. H. Harsant, 113; W. J. Penny, 201; Dr. J. Swain, 297.
Surgical handicraft—W. Pye (*Review*), 218.
Surgical practice, Golden rules of (*Review*), 53.
Swain, Dr. J.—Report on surgery, 297.
Swayne, Dr. J. G.—Forceps delivery during the last fifty years, 153.
Symphysiotomy, 306.
Syphilis in ancient and prehistoric times—Dr. F. Buret (*Review*), 220.

Tenby as a health-resort — J. G. Lock, 97.
Tetanus, 295.
Thin, Dr. G.—Leprosy (*Review*), 129.
Throat, nose, and ear diseases—Dr. P. McBride (*Review*), 137.
Treves, F.—A manual of operative surgery (*Review*), 45.
Tuberculosis, 294.
Tuckey, Dr. C. L.—Psycho-therapeutics (*Review*), 132.
Typhoid fever, 293; Treatment of—Dr. J. B. Yeo (*Review*), 50; Dr. J. Barr (*Review*), 214.

Ureter, Calculus in, 32.
Urethra, Stricture of the female, 31.

Voice-training, 126.

Water cure in the bedroom—Dr. G. H. Doudney (*Review*), 134.
Williams, Dr. P. W.—Hay-fever, 84.
Women, Diseases of—Dr. H. M. Jones (*Review*), 49; Dr. A. H. N. Lewers (*Review*), 219.
Wry-neck, Spasmodic—N. Smith (*Review*), 51.

Year-book of scientific societies (*Review*), 318.
Year-book of treatment (*Review*), 52.
Yeo, Dr. J. B.—Treatment of typhoid fever (*Review*), 50.

### *PUBLICATIONS RECEIVED.*

From ADLARD & SON, London:
*Four Hundred Cases of Phthisis.* By F. M. SANDWITH, M.D. 1892.

From BAILLIÈRE, TINDALL and COX, London:
*A Guide to the Examinations in Sanitary Science.* By HERBERT JONES. 1892.

From BAISS BROTHERS & CO., London:
*The Quarterly Therapeutic Review.* Vol. X., No. 40. October, 1892.

From JOHN BALE & SONS, London:
*The Operative Treatment of Enlargement of the Prostate.* By C. W. MANSELL MOULLIN. 1892.

From L. BRUCK, Sydney:
*The Australasian Medical Directory.* 1892.

From BURROUGHS, WELLCOME & CO., London:
*A B C Medical Diary and Visiting List for* 1893.
*From "Oesypum" to "Lanoline."* 1892.

From CASSELL & COMPANY, LIMITED, London:
*Advice to Women on the Care of their Health, before, during and after Confinement.* By FLORENCE STACPOOLE. 1892.
*Our Sick: and How to Take Care of Them.* By FLORENCE STACPOOLE. 1892.

From J. & A. CHURCHILL, London:
*Eyestrain.* By ERNEST CLARKE, M.D. 1892.
*Rome and Malaria.* By Professor TOMMASI-CRUDELI. 1892.
*Reform in Treatment of the Insane.* By D. HACK TUKE, M.D. 1892.
*Antiseptic Dry-Air Treatment of Consumption.* By JOHN J. HARTNETT, M.D. Second Edition. 1892.
*The Value of Hypnotism in Chronic Alcoholism.* By C. LLOYD TUCKEY, M.D. 1892.
*Manual of Orthopœdy: The Head and Neck.* By HEATHER BIGG. 1892.

From THE CLARENDON PRESS, Oxford:
*Epidemic Influenza.* By F. A. DIXEY, M.D. 1892.

From T. & A. CONSTABLE, Edinburgh:
*Atlas of Clinical Medicine.* By BYROM BRAMWELL, M.D. Vol. II., Part I. 1892.

From THE F. A. DAVIS CO., Philadelphia.
*Book on the Physician Himself.* By D. W. CATHELL, M.D. Tenth Edition. 1892.
*Diseases of the Lungs, Heart, and Kidneys.* By N. S. DAVIS, jun., A.M., M.D. 1892.

From FERET ET FILS, Bordeaux:
*I.—Abcès du Larynx dans la Scarlatine. II.—Un Nouveau Cas de Chancre Induré de la Fosse Nasale.* Dr. E. J. MOURE. 1892.

From CHARLES GRIFFIN & COMPANY, LIMITED, London:
*The Diagnosis of Diseases of the Heart and Thoracic Aorta.* By A. ERNEST SANSOM, M.D. 1892.

From JOHN HEYWOOD, Manchester:
*Transactions of the Pathological Society of Manchester for the Session* 1891–92. Edited by T. N. KELYNACK, M.B. Volume the First.

From H. H. HOWE, Weston, Vt., U.S.A.:
*The Abstract and Index.* September, October, 1892.

From B. Keen, Bristol:
*The Health Messenger.* No. 15. October 15th, 1892.

From E. Knight, London:
*The Clinical Journal.* Vol. I., Nos. 1—6. Nov. 2nd—Dec. 7th, 1892.

From J. W. Leng, London:
*The Medical Pioneer.* Nos. 1—3. October—December, 1892.

From H. K. Lewis, London:
*Epidemic Skin Disease.* By Thomas D. Savill, M.D. 1892.
*The British Journal of Dermatology.* October—December, 1892.
*Chart for Recording the Examination of Urine.*
*Hygiene.* By Louis C. Parkes, M.D. Third Edition. 1892.
*Diseases of the Eye.* By Henry R. Swanzy, M.B. Fourth Edition. 1892.
*Notes on the Clinical Examination of the Blood and Excreta.* By Sidney Coupland, M.D. Third Edition. 1892.

From Sampson Low, Marston & Company, London:
*Cocoa: All About It.* By "Historicus." 1892.

From J. H. Musser, M.D., Pennsylvania.
*Tuberculous Ulcer of the Stomach.* By J. H. Musser, M.D. 1890. [Reprint.]
*Grave Forms of Purpura Hæmorrhagica.* By J. H. Musser, M.D. 1891. [Reprint.]
*The Limitations and the Powers of Therapeutics.* By J. H. Musser, M.D. 1892. [Reprint]

From Owles & Reader, London:
*The Book Review Index.* June, 1892.

From Young J. Pentland, Edinburgh:
*Disease in Children.* By James Carmichael, M.D. 1892.
*Pronouncing Dictionary of Medical Terms.* By John M. Keating, M.D. 1892.
*Malignant Disease of the Throat and Nose.* By David Newman, M.D. 1892.
*The Journal of Pathology and Bacteriology.* Vol. I., Nos. 1, 2. May, October, 1892.

From John Morgan Richards, London:
*Medical Reprints.* September—November, 1892.

From Rigaud & Chapoteaut, Paris:
*The Salts of Strontium.* Paraf-Javal. 1892.

From G. P. Roy & Co., Calcutta:
*The Scalpel.* Vol. I., Nos. 1, 2. October, November, 1892.

From The Royal Meteorological Society, London:
*English Climatology,* 1881–1890. By Francis Campbell Bayard. [Reprint.]
*The Value of Meteorological Instruments in the Selection of Health Resorts.* By C. Theodore Williams. [Reprint.]

From J. P. Segg & Co., London:
*How to Give Gas.* By T. E. Constant. [1892.]

From J. Shepherd Clark Co., New York:
*La Revista Médico-Quirúrgica Americana.* Vol. I., No. 1. Septiembre, 1892.

From Whittaker & Co., London:
*Dissections Illustrated.* By C. Gordon Brodie. Part I. 1892.

From John Wright & Co., Bristol:
*Golden Rules of Surgical Practice.* By E. Hurry Fenwick. Third Edition. [1892.]

From J. Young & Sons, Perth:
*The Sixty-fifth Annual Report of James Murray's Royal Asylum, Perth.* 1892.

